# Supplementary material for: Mendelian Randomization Reveals: Triglycerides and Sensorineural Hearing Loss
Source: Bioengineering (Basel). 2024 Apr 29;11(5):438. doi: 10.3390/bioengineering11050438 (PMC11118253; doi:10.3390/bioengineering11050438)
Supplement: Supplementary file 1 [file bioengineering-11-00438-s001.zip › bioengineering-2955688-supplementary/Supplementary materials/Supplementary Figures.pdf]

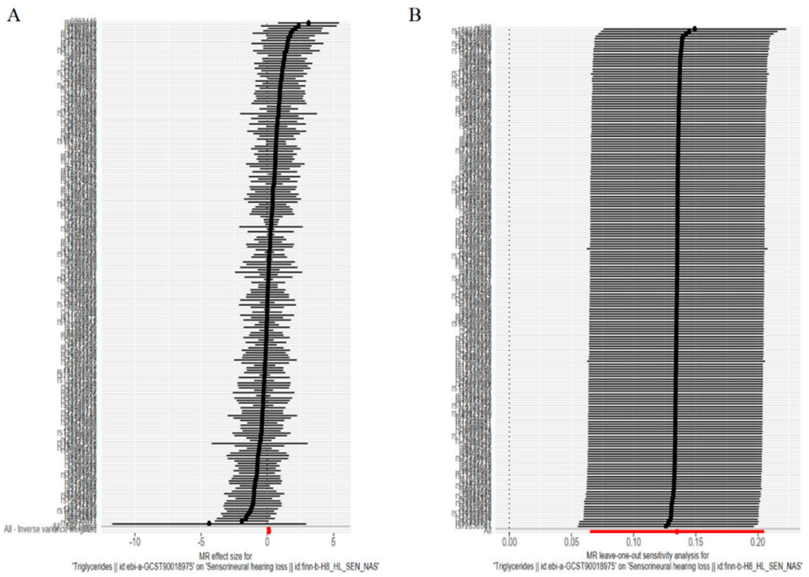

Figures S1 Related results in the initial MR analysis:(A) Forest plots illustrating the MR results for TG and SNHL; (B) Leave-one-out analysis of the MR results for TG and SNHL.

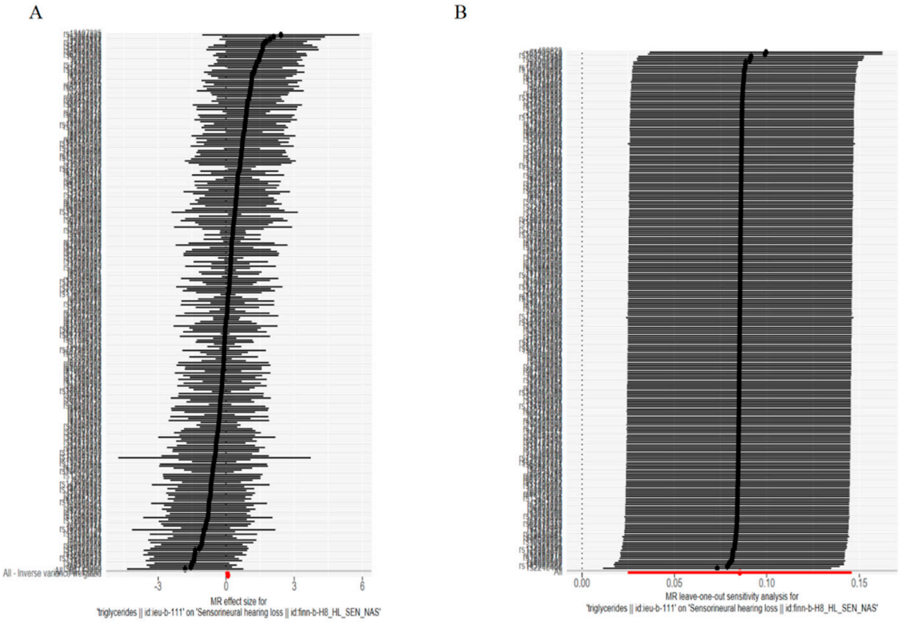

Figures S2 Related results in the initial MR analysis:(A) Forest plots illustrating the MR results for TG and SNHL; (B) Leave-one-out analysis of the MR results for TG and SNHL.
