# Supplementary material for: Mendelian Randomization Reveals: Triglycerides and Sensorineural Hearing Loss
Source: Bioengineering (Basel). 2024 Apr 29;11(5):438. doi: 10.3390/bioengineering11050438 (PMC11118253; doi:10.3390/bioengineering11050438)
Supplement: Supplementary file 1 [file bioengineering-11-00438-s001.zip › bioengineering-2955688-supplementary/Supplementary materials/Supplementary File S1.pdf]

Table S1 Basic information of the GWAS database in the two-sample MR study

Name	ID	Sample size	Race	Number of SNPs	Year
TG	ebi-a-GCST90018975	343992	European	19052580	2021
TG	ieu-b-111	441016	European	12321875	2020
SNHL	finn-b-H8_HL_SEN_NAS	212544	European	16380454	2021
